# Human Immune Responses to Dengue Virus Infection: Lessons Learned from Prospective Cohort Studies

**DOI:** 10.3389/fimmu.2014.00183

**Published:** 2014-04-24

**Authors:** Timothy P. Endy

**Affiliations:** ^1^Infectious Disease Division, Department of Medicine, State University of New York Upstate Medical University, Syracuse, NY, USA

**Keywords:** dengue virus, prospective cohort studies, lessons, vaccine, development

## Abstract

Dengue virus (DENV) continues to spread globally and is a major cause of morbidity and mortality. Currently, there is no antiviral treatment to diminish severe illness or a vaccine to induce protection from infection. An effective dengue vaccine that protects against all four DENV serotypes is a high priority for endemic countries and several candidates are in development by various United States Federal Agencies and private pharmaceutical companies. Challenges faced by dengue vaccine developers include creating tetravalent formulations that provide tetravalent protection, the lack of a correlate of protective immunity, a changing viral landscape as DENV evolves, and a complex viral-host pathogenesis that can result in a spectrum of illness from subclinical infection to severe hemorrhagic fever. There have been a number of long-term prospective studies on DENV transmission and dengue severity that have provided invaluable information on DENV epidemiology and pathogenesis of this disease. In this section, we will review the critical lessons learned from these studies and their application for dengue vaccine development.

## Introduction

The global dengue pandemic and its associated morbidity and mortality have been covered in other excellent reviews and sections of this textbook and will not be reviewed here. Prospective studies have been a valuable tool in understanding the epidemiology and pathogenesis of dengue virus (DENV) infection. For the dengue vaccine developer specifically these studies offer the advantage of determining the true incidence of infection, the full spectrum of clinical outcomes from subclinical to severe hospitalized illness, risk factors for disease severity, and viral information on the genetics and evolution of DENV and its spatial and temporal spread. Recently, the results of a Phase 2b candidate tetravalent DENV vaccine [yellow fever (YF)-dengue chimeric, Sanofi Pasteur] were published (1). This was conducted in a highly flavivirus antibody experienced cohort of children in Thailand and demonstrated an excellent safety and neutralizing antibody immunogenicity profile. The vaccine however, failed to achieve protection against all four DENV types with an overall efficacy of 30.2%. Efficacy varied by DENV type with the lowest noted against DENV-2 (9.2%), which was the predominant circulating DENV serotype at the time of the trial. The results of this efficacy trial highlighted several development challenges for a dengue vaccine including the lack of a correlation of protection despite the detection of serotype-specific neutralizing antibody.

In this section, the lessons learned from the prospective cohort studies and their application to DENV vaccine development will be reviewed. Though much information has been learned from these important studies, there are six important lessons learned that I believe are critical for DENV vaccines and include: (1) incidence rates will support Phase III efficacy studies though a high degree of temporal and spatial diversity in incidence occurs requiring consideration in choosing populations; (2) demonstration that pre-existing serotype-specific neutralizing antibody does not protect against infection suggests that our current assays to measure neutralizing antibody are not a correlate for protection; (3) the symptomatic to inapparent (S:I) ratio is an epidemiologic correlate for heterologous protective immunity or enhancement and may be a correlate for vaccine efficacy; (4) the time from last infection determines the S:I ratio and provides evidence of a half-life of heterologous protective immunity, which may affect the observed efficacy in DENV vaccines depending on the time point efficacy is measured; (5) there is a high degree of temporal and spatial genetic diversity in the DENVs reinforcing the need for a tetravalent DENV vaccine but also consideration for the need of genotypic-specific protection as well; (6) changing population dynamics and viral evolution with older individuals becoming infected suggest that immunosenescence and potential viral escape mutants should be considered when formulating DENV vaccines.

## Prospective Cohort Studies

Prospective cohort studies on DENV transmission; the surveillance of a select group of individuals over time for DENV infection, are an invaluable source of information on the incidence of infection, viral, and host factors that lead to subclinical infection or severe disease, and understanding the full burden of DENV infection. For testing DENV vaccines, they are essential in determining the true efficacy of a vaccine. Table 1 is a summary of the published prospective dengue cohort studies to date. The first prospective cohort study was conducted in Rayong, Thailand in January 1980 among children who were sampled from schools and households (2). Pre- and post-epidemic cohort blood samples determined that the incidence of dengue infection in 251 seronegative children was 39.4%. The average incidence of hospitalized dengue was 0.7%. Of the shock syndrome cases admitted to the hospital, the major risk factor for severe infection was secondary infection. The Rayong study was the first to establish the high burden of dengue illness in Thailand, the association of secondary dengue infections with severe dengue illness, the circulation of all four DENV serotypes and the preponderance of one specific serotype in a given epidemic year, and the importance of sequential dengue serotypes in producing shock syndrome. Despite the variation in incidence amongst the prospective studies listed in Table 1, the incidence of severe hospitalized disease is remarkable consistent, all countries where they were conducted have a high burden of DENV infection and >80% of severe infections are associated with subsequent DENV infections after a primary infection.

**Table 1:** **Summary of prospective cohort studies of DENV transmission and disease**.

Study site	Population size^a^	Age range (years)	Study period	Incidence (average)				
				Dengue infection (%)	Symptomatic dengue	Hospitalized dengue	Severe dengue	Symptomatic: asymptomatic ratio
Rayong, Thailand (2)	1,056	4–14	1980–1981	39.4	n/a^b^	0.7%	0.7%	n/a
Bangkok, Thailand (3)	1,757	4–16	1980–1981	11.8	0.7%	0.4%	0.4%	1:8
Yangon, Myanmar (4)	12,489	1–9	1984–1988	5.1	n/a	0.3%	0.2%	n/a
Yogyakarta, Indonesia (5)	1,837	4–9	1995–1996	29.2	0.6%	0.4%	0.4%	n/a
Kamphaeng Phet I, Thailand (6)	2,119	7–11	1998–2002	7.3	3.9%	1.0%	0.6%	1:0.9
Iquitos, Peru (7)	2,300	5–20	1999–2005	34.5	n/a	n/a	n/a	n/a
West Java, Indonesia (8)	2,536	18–66	2000–2002	7.4	1.8%	0.1%	0.1%	1:3
Managua, Nicaragua (9, 10)	1,186	4–16	2001–2002	9.0	0.85%	n/a	n/a	1:13–1:6
Maracay, Venezuela (11)	981	5–13	2001–2002	16.9	n/a	n/a	n/a	n/a
Kamphaeng Phet II, Thailand (12)	2,095	4–16	2004–2006	6.7	2.2%	0.5%	0.1%	1:3.0
Ratchaburi, Thailand (13, 14)	3,015	3–11	2006–2009	3.6	3.6%	1.6%	0.3%	n/a
Managua, Nicaragua (10)	3,800	2–9	2004–2010	9.0	0.85%	n/a	n/a	1:13–1:6
Long Xuyen, Vietnam (15)	2,190	2–15	2004–2007	3.0	3.0%	1.2%	1.2%	n/a
Southeast Asia (Indonesia, Malaysia, Philippines, Thailand, and Vietnam (16)	1,500	2–14	2010–2011	11.4	n/a	n/a	n/a	n/a

*^a^Number in cohort tested for dengue antibody (incidence denominator)*.

*^b^n/a, not available; not provided in the published paper*.

The second prospective study was a 2-year (1980–1981) school-based study involving 1,757 children, ages 4–16 years, in Bangkok, Thailand (3). This was the first study to use active surveillance using school absence as an indicator of potential illness. Antibody titer revealed that 50% of the enrolled students had evidence of dengue antibody, likely indicative of a DENV infection experienced prior to the age of 7 years, and the first to demonstrate that even at this young age the population is already highly flavivirus antibody experienced. Most (87%) of the students who became infected during the study period were asymptomatic as determined by lack of clinical illness. Important study findings were an incidence in dengue-naïve participants of 6.3%, in dengue-experienced participants an incidence of 5.5% and among symptomatic infected children a hospitalization rate of 53%. The symptomatic-to-asymptomatic ratio of DENV infection was 1:8. This study confirmed the importance of subsequent DENV infection as a risk factor for severe infection with an odds ratio for developing DHF in participants with pre-existing dengue immunity ≥6.5. The importance of this prospective study was as the first study to determine the full burden of DENV infection within a cohort, the incidence of infection and the relationship of pre-existing DENV immunity to dengue disease severity.

In the last decade a number of prospective cohort studies on DENV transmission have been performed adding to our knowledge on DENV incidence and risk factors for severe infection. These include an ongoing study in Iquitos, Peru (the first in the Americas), one in Indonesia (the first in adults), an ongoing study in Nicaragua, studies in Venezuela, Vietnam, Malaysia, Philippines, Vietnam, and ongoing studies in Kamphaeng Phet, Thailand.

## Incidence Rates will Support Phase III Efficacy Studies Though a High Degree of Temporal and Spatial Diversity in Incidence Occurs Requiring Consideration in Choosing Populations

The prospective studies from both the America and Asia have demonstrated a high burden of DENV infection and incidence rates that clearly support economically feasible DENV vaccine efficacy studies. With an average incidence of symptomatic DENV infection of 2%, a sample size calculation using an 80% efficacy of the vaccine, an alpha value of 0.05, power of 0.8, will require a total sample size of vaccinated and controls of 1,452 volunteers. Studies in Iquitos, Peru and in Kamphaeng Phet, Thailand (referenced in Table 1) demonstrated the heterogeneity of DENV incidence and serotype-specific transmission spatially and temporally and illustrated elegantly in schools in Kamphaeng Phet, Thailand. In Kamphaeng Phet, dengue incidence and serotype-specific transmission was cyclical in each school, with relatively mild years followed by more severe years. A proportion of schools had a severe dengue year while other schools a short distance away had less severe dengue. Similarly, one school would have a DENV-2 outbreak and another a short distance away a DENV-3 outbreak. The diversity of incidence both spatially and temporally is an important observation from these studies in designing dengue vaccine efficacy studies and in estimating and choosing the population and geographic location required to assess statistical efficacy.

## Pre-Existing Serotype-Specific Neutralizing Antibody Does Not Protect Against Infection Suggests That Our Current Assays to Measure Neutralizing Antibody is Not a Correlate for Protection

Historically and currently, DENV neutralizing antibody has been a serologic biomarker to measure previous and current DENV serotype-specific infection (1, 17, 18). The role of homotypic and heterotypic antibody as a marker of immunity was first described in studies performed by Sabin in the 1940s in human volunteers (19, 20). The concept as demonstrated was that following a serotype-specific DENV infection, homotypic antibody develops to that specific serotype that last life-long and becomes a biomarker that indicates previous infection to that serotype and thus durable immunity. Heterotypic, cross-reactive antibody to other DENV serotypes following a serotype-specific infection also develops, is transient, and may modify disease severity when infected with another serotype. The development of the plaque reduction neutralization titer (PRNT) assay and its variation for DENV became a relatively high-throughput assay to measure this biomarker for previous and current serotype-specific protection (17). As such it has evolved to be an important biomarker to measure DENV vaccine performance in pre-clinical and clinical DENV vaccine trials. We have demonstrated that this assay has a high degree of variability in its performance depending on the cell lines and prototype viruses used and the detection of heterotypic neutralizing antibody creates confusion in its interpretation of whether an individual is protected or not from a specific serotype (21). In the Kamphaeng Phet cohort studies, the active surveillance for infection and analysis of archived sera from infected individuals allowed the examination of serotype-specific PRNT antibody just prior to severe DENV infection (22). What was demonstrated was the observation that individuals hospitalized for severe serotype-specific DENV had detectable PRNT titers to that serotype using either prototype DENV and to their own isolated virus approximately 6 months prior to infection. The implication of this finding was that the PRNT was not an adequate biomarker for serotype-specific protection. Ten years later this finding was reiterated during the vaccine efficacy studies of the ChimeriVax vaccine (1). Despite high levels of DENV-2 neutralizing antibody as detected by PRNT, only 9.2% were protected. These findings highlight the need for the development of biomarkers that are highly predictive of serotype-specific DENV protection and currently a major obstacle in the development of DENV vaccines.

## Symptomatic to Inapparent Ratio is an Epidemiologic Correlate for Heterologous Protective Antibody and may be a Correlate for Vaccine Efficacy

The original Bangkok prospective study demonstrated the full burden of DENV infection, documented the occurrence of subclinical infection and introduced the concept of the S:I ratio (3). In the Kamphaeng Pet cohort studies, we have demonstrated that the S:I ratio is not a fixed variable but there is a variance in the ratio that varies spatially and temporally (6). This ratio was fluid with schools experiencing more symptomatic disease, high S:I ratio, than other schools which shifted the following year where schools had more subclinical infections, lower S:I ratios, while other schools experienced high S:I ratios. This variation in the S:I ratio when analyzed closely is not a random event but occurs in a cyclic pattern with one school having a high S:I ratio, more symptomatic infection, followed in the same school by years of lower S:I ratios, less symptomatic infection. This pattern was reflected at the individual school level and also as a population as a whole. An explanation of this variance is that the S:I ratio is an epidemiologic correlate for an undercurrent of heterotypic protective immunity that may not prevent infection but may modify disease severity. At an individual level what this implies is that following a serotype-specific infection there is the generation of heterotypic antibody that modulates severity of infection that fades with time. At the same time there is a heterotypic enhancing antibody that can influence the production of more severe dengue infection. Expanding from an individual level, this effect applies to local populations such as schools, villages and to the population as a whole and will be discussed in more detail in the next section. The data suggest that the S:I ratio is an epidemiologic correlate for this protective or disease enhancing heterotypic antibody and reflects the time-dependant flavivirus experience of a population. For DENV vaccine studies, the S:I ratio may be a very important correlate of the vaccine’s ability to confer heterotypic protection or enhancement of disease severity and potentially an important efficacy correlate to follow over time in a population.

## Time from Last Infection Determines the S:I Ratio and Provides Evidence of a Half-Life of Heterologous Protective Immunity Which may Affect the Observed Efficacy in DENV Vaccines Depending on the Time Point Efficacy is Measured

As discussed, the S:I ratio in a population over time is an epidemiologic correlate that reflects an undercurrent of both heterotypic protective and enhancing immunity that is time-dependant. We and others have demonstrated that the time from last infection determines the S:I ratio and that there is a measurable half-life of heterologous protective immunity (23, 24). This was elegantly demonstrated in the Kamphaeng Phet cohort studies and recently published (23). Data from the prospective cohort studies were analyzed for subclinical and symptomatic DENV infections in schoolchildren from 1998 to 2007. Children who experienced one or more DENV infection were selected as the population for analysis (2,169 person-years of follow-up). Demonstrated from the analysis was a shorter time interval between infections was associated with subclinical infection in children who were seronegative for DENV at enrollment; seronegative being an important pre-existing condition to document primary to secondary infections as compared to the more complicated second to third infections. This time interval was an average of 1.6 years from first infection that resulted in a subclinical infection, 1.9 years for symptomatic dengue fever (DF), and 2.6 years for the onset of severe hospitalized dengue hemorrhagic fever (DHF). These findings indicate that there is a window of cross-protection following DENV infection that last approximately 1.6 years following infection. The implications of these findings for DENV vaccine efficacy studies are subtle but important. The goal for a tetravalent DENV vaccine is to confer durable tetravalent DENV immunity and protection against severe illness. If one serotype in the DENV tetravalent formulation in particular induces a brisk immune response, the results from the prospective cohort studies would suggest that there will be heterotypic protective immunity from that one serotype to other serotypes conferring tetravalent protection that lasts for 1.6 years. If true, then measuring efficacy at 1 year following vaccination as the most recent DENV vaccine trial did, would not be a true measurement of tetravalent protection. A more appropriate efficacy time point would be at least 3 years following vaccination as a true measurement of efficacy. This of course would mean a longer duration of observation of the population following vaccination and cost of the study.

## There is a High Degree of Temporal and Spatial Genetic Diversity in the DENVs Reinforcing the Need for a Tetravalent DENV Vaccine but also Consideration for the Need of Genotypic-Specific Protection as Well

It is thought that the DENV transmission evolved into four distinct serotypes approximately 1,000 years ago and each of these four serotypes emerged into a cycle of transmission between humans and its mosquito vector approximately 125–320 years ago (25, 26). Phylogenetic analysis suggests that the DENVs are rapidly evolving with major clade replacements and genetic shifts occurring in populations endemic for DENV (25–28). Asia has been pivotal in the evolution of DENV as the location of the first cases of the more severe form of DENV infection, DHF, which made its first appearance in the 1950s first in the Philippines then in Thailand (29). The current Asian genotypes of each serotype are considered more severe, result in more severe dengue illness, than the American genotypes (30). Evidence suggests that DENV circulation in Asia due to its population growth and urbanization, high vector burden, and high level of pre-existing flavivirus seroprevalence, contribute to the increase in genetic diversity which is estimated as increasing at a factor between 14 and 20 in the last 30 years (31). The overall impression of DENV evolution in Asia is the active transmission of viruses in individuals who are highly flavivirus antibody experienced causing evolutionary pressure on the virus to evolve to escape and utilize pre-existing flavivirus immunity. By its nature the current evolving DENVs are adept at escaping heterologous neutralizing antibody and using it as a means to attain high viral load levels and more severe disease through antibody enhancement.

To understand the evolution and phylogeography of DENV, the prospective cohort studies in Kamphaeng Phet have been isolating and sequencing DENVs from individuals in specific geographic areas and over time. In ongoing studies, viruses are isolated and through the use of global positioning system (GPS) the exact geographic position located allowing specific spatial and temporal analysis. Analysis of isolated viruses demonstrated diverse viral genetic variation in both time and space in the Kamphaeng Phet population with multiple viral lineages circulating within individual schools and villages (32). This implies that there is frequent gene flow of DENV into this microenvironment as individuals move from major urban areas into smaller village. At the microenvironment level, there was clustering of specific viral genotypes within individual schools that are highly conserved from year to year and evidence of frequent viral gene flow among schools closely related in space and time. These results suggest that there is a combination of frequent viral migration into Kamphaeng Phet coupled with population subdivisions of conserved viruses that shape the genetic diversity of DENV at a local and population scale. Taken together is a picture of a virus that is evolving rapidly, spread by population movement, and due to the restricted flight of the vector, genetically conserved at a microenvironment level.

For a DENV vaccine these findings have many implications with the most important is that the current vaccines using historical prototype viruses collected in the 1980s may no longer provide the protection to current circulating DENV genotypes and raises the question if protection needs to be both serotype and genotype specific. This question was raised during the ChimeriVax vaccine trial where the DENV-2 vaccine strain did not match the circulating wild-type DENV-2 and failed to produce protection (1).

## Changing Population Dynamics and Viral Evolution with Older Individuals Becoming Infected Suggest That Immunosenescence and Potential Viral Escape Mutants Should be Considered When Formulating DENV Vaccines

Dengue illness in endemic countries is considered to be primarily a pediatric disease due to the high degree of transmission and infection that occurs at a young age. Adults are considered highly flavivirus experienced and thus protected from subsequent dengue illness. For this reason, the prospective cohort studies have largely been performed in pediatric populations and illness criteria of severity of disease developed in pediatric populations. In our own cohort studies, we have noted that the age of symptomatic DENV infection has been increasing slowly over the last decade by a median increase of 2–4 years. This has been reflected in Thailand as a whole and interestingly in the rest of Southeast Asia (33). An analysis and mathematical modeling of this phenomenon was performed on data from each of the 72 provinces of Thailand (33). What was described was that the force of infection has declined by 2% each year with the strongest predictor of this change the median age of the population, which was reflective of the changing demographics of the population. The authors’ conclusion was that lower birth and death rates decrease the flow of susceptible individuals into the population and thereby increase the longevity of immune individuals. For a DENV vaccine, the implications are despite the decrease in the force of infection the critical vaccination fraction has not changed declining from 85 to 80% (2). A key point in this argument however is the longevity of immune individuals and if protection is life-long. Recently, in our own studies in Kamphaeng Phet we have been documenting a large number of adults who are older than 50 years of age with one 80-year old hospitalized with severe DENV infection. These are individuals who have never moved from Kamphaeng Phet, a highly endemic region for DENV, and defy statistical probability of never having been infected by one or more DENVs in their previous experience. This is under active investigation but does suggest that the aging immune system, immunosenescence, might be a factor in older individuals becoming ill from DENV infection or, and particularly worrisome, that the current strains/genotypes have evolved as escape mutants and individuals are no longer protected from current wild-type DENV strains/genotypes despite infection from another genotype but same serotype in the past. This as noted in the previous section has implications on the current DENV vaccine candidates and whether their vaccine prototype strains/genotypes are applicable to current circulating genotypes.

## Summary

The prospective studies have been invaluable in increasing our understanding on the epidemiology and pathogenesis of this globally important virus and disease. Discussed are important lessons learned from these studies that have direct application to current DENV vaccine development and testing as they move forward in their regulatory development to a licensed vaccine. Identified are critical issues that are challenges for the development of an effective tetravalent DENV vaccine. Additional research is needed to address these areas that are critical to vaccine testing and evaluation. Expanding cohort studies to include those countries where dengue is inadequately characterized is critical for us to further understand the unique viral and host factors that contribute to differences in dengue risk and vaccine response.

## Conflict of Interest Statement

The author declares that the research was conducted in the absence of any commercial or financial relationships that could be construed as a potential conflict of interest.

## Abbreviations

DENV, dengue virus; DF, dengue fever; DHF, dengue hemorrhagic fever; NAb, neutralizing antibody; PRNT, plaque reduction neutralizing titer.
